# Autophagic Removal of Farnesylated Carboxy-Terminal Lamin Peptides

**DOI:** 10.3390/cells7040033

**Published:** 2018-04-23

**Authors:** Xiang Lu, Karima Djabali

**Affiliations:** Epigentics of Aging, Department of Dermatology, TUM School of Medicine, Technical University of Munich, 85748 Garching-Munich, Germany; x.lu@tum.de

**Keywords:** progerin, nuclear envelope, autophagy, prelamin A, SUN1, farnesylation

## Abstract

The mammalian nuclear lamina proteins—prelamin A- and B-type lamins—are post-translationally modified by farnesylation, endoproteolysis, and carboxymethylation at a carboxy-terminal CAAX (C, cysteine; a, aliphatic amino acid; X, any amino acid) motif. However, prelamin A processing into mature lamin A is a unique process because it results in the production of farnesylated and carboxymethylated peptides. In cells from patients with Hutchinson–Gilford progeria syndrome, the mutant prelamin A protein, progerin, cannot release its prenylated carboxyl-terminal moiety and therefore remains permanently associated with the nuclear envelope (NE), causing severe nuclear alterations and a dysmorphic morphology. To obtain a better understanding of the abnormal interaction and retention of progerin in the NE, we analyzed the spatiotemporal distribution of the EGFP fusion proteins with or without a nuclear localization signal (NLS) and a functional CAAX motif in HeLa cells transfected with a series of plasmids that encode the carboxy-terminal ends of progerin and prelamin A. The farnesylated carboxy-terminal fusion peptides bind to the NE and induce the formation of abnormally shaped nuclei. In contrast, the unfarnesylated counterparts exhibit a diffuse localization in the nucleoplasm, without obvious NE deformation. High levels of farnesylated prelamin A and progerin carboxy-terminal peptides induce nucleophagic degradation of the toxic protein, including several nuclear components and chromatin. However, SUN1, a constituent of the linker of nucleoskeleton and cytoskeleton (LINC) complex, is excluded from these autophagic NE protrusions. Thus, nucleophagy requires NE flexibility, as indicated by SUN1 delocalization from the elongated NE–autophagosome complex.

## 1. Introduction

The nuclear envelope (NE) is an extension of the endoplasmic reticulum (ER), and comprises the outer nuclear membrane (ONM) and the inner nuclear membrane (INM) [1]. The ONM is enriched in ER components; however, the INM contains numerous distinctive membrane proteins, including the LEM-domain protein emerin and the KASH-domain protein, SUN1 [2]. The NE double membranes are separated by a perinuclear space (PNS) and only intermingle at the joint site at which nuclear pore complexes (NPCs) are located [3,4]. NPCs are bidirectional transporters, shuttling macromolecules between the cytoplasm and the nucleus and vice versa [4,5]. The nuclear lamina underlies the INM and was originally considered a mechanical support for the nucleus, but its function has further been extended to signal transduction through interactions with INM proteins and genome stability via chromatin binding [6]. 

Nuclear lamins are members of the intermediate filament protein (IFP) family [7]. Mammalian cells express two types of lamins, A- and B-types, which are encoded by *LMNA*, *LMNB1*, and *LMNB2* genes, respectively [8,9]. Like cytoplasmic IFP proteins, A- and B-type lamins share a similar structure that comprise an N-terminal domain head domain (NT), a central a rod domain, and a C-terminal tail domain (CT) [10]. A CaaX motif (C, cysteine; a, aliphatic amino acid; X, any amino acid) with an exact sequence of CSIM is present at the C-terminus in all B-type lamins and the lamin A precursor, known as prelamin A [11]. Prelamin A (preLA) undergoes multiple steps of post-translational modification (PTM) at its C-terminus and eventually releases the mature lamin A protein, which integrates into the nuclear lamina [12]. The CaaX motif is a signal for prenylation, resulting in the attachment of a farnesyl group to its cysteine residue and subsequent cysteine farnesylation [13,14]. Following farnesylation, the removal of the aaX residues by the Rec 1 or Zmpste 24 endoprotease induces carboxymethylation of the protein [12]. Then, the removal of the last 15 amino acids from the C-terminal by Zmpste 24 produces the mature lamin A [12,15]. The *LMNA* G608G mutation associated with Hutchinson–Gilford progeria syndrome (HGPS) is a dominant negative mutation in exon 11 of *LMNA* gene that creates an alternatively spliced mRNA isoform, resulting in a 50-amino acid (a.a.) in-frame deletion of preLA at its carboxy-terminal domain, termed progerin [16,17]. Progerin remains permanently farnesylated and carboxymethylated at its C-terminus due to absence of the Zmpste 24 cleavage site [18,19]. Based on the current state of HGPS research, farnesylated progerin is toxic to cells and causes the mutant protein to remain anchored to the nuclear membrane. This nuclear localization disrupts the underlying lamina in a dominant negative fashion and leads to all of the downstream nuclear defects that are characteristic of HGPS, such as nuclear blebbing, heterochromatin disorganization, mislocalization of nuclear envelope proteins, and disrupted gene transcription [20]. An ultrastructural analysis of the nuclei of HGPS cells showed alterations in chromatin organization, with a loss of heterochromatin at the nuclear envelope periphery and an increased number of nuclear envelope invaginations with clustering of the nuclear pores (NPCs) [21,22,23]. Progerin expression also induces alterations in the composition of the nuclear lamina, with loss of lamin B1 and modification in NE transmembrane protein levels and distribution at the NE [24,25]. All these progerin-induced changes in the nuclear lamina—an architectural meshwork that determines the size, shape, and functional properties of the nucleus—apparently affect fundamental processes including proliferation, differentiation, and premature senescence [26]. The mechanism by which progerin directly contributes to the pathology of HGPS is not completely understood. However, the tight connection between progerin and SUN1, an INM component of the LINC (linker of nucleoskeleton and cytoskeleton) complex that connects the nuclear lamina to the cytoskeleton, might contribute to the structural changes in the NE and the endoplasmic reticulum (ER) in HGPS cells [27]. Moreover, concomitantly with the accumulation of progerin in HGPS cells, SUN1 levels are also increased [27,28]. The SUN1–progerin interaction appears to depend on the permanent farnesylation of progerin causing both proteins to remain associated within the ER during mitosis [27,29]. Farnesylation of progerin enhances its interaction with SUN1 and reduces SUN1 mobility, thereby promoting the aberrant recruitment of progerin to the ER membrane during postmitotic assembly of the NE. This results in the accumulation of SUN1 over consecutive cell divisions [27,29]. 

In this study, we investigated the impact of the farnesylated carboxy-terminal ends of progerin and prelamin A on the nuclear envelope morphology and the localization of SUN1, emerin, lamin A/C and lamin B1, as well as the nuclear pores. We also tracked the subcellular localization of overexpressed farnesylated EGFP fusion proteins to determine the storage sites and/or degradation pathway. 

## 2. Materials and Methods

### 2.1. Plasmid Constructs

The full-length prelamin A and progerin cDNA were inserted into the pEGFP-c1 vector (Clontech, Fremont, CA, USA). Protein residues 602–664 a.a. from the C-terminus of prelamin A linked to a classical nuclear localization signal (NLS, KKRKLLE) were designed as NLS-59-CSIM. The NLS-59-R-CSIM containing the L647R mutation and NLS-59-SSIM containing the C661S mutation were designed as corresponding mutant isoforms. The in-frame deletion of 50 a.a. from residues 607–656 of prelamin A were designed as NLS-50 and ΔNLS-50; these constructs either contained or lacked the NLS, respectively. Residues 641–664 a.a. of prelamin A linked to NLS were designed as NLS-20-CSIM. The NLS-20-R-CSIM and NLS-20-SSIM corresponded to L647R and C661S mutant of NLS-20-CSIM. The C-terminal residues 602–614 of progerin were linked to the NLS to create NLS-PG-9-CSIM. The corresponding mutant isoform was NLS-PG-9-SSIM. The PG-9-CISM and PG-9-SSIM isoforms were designed to determine the cellular distribution of progerin C-terminal fragments lacking the NLS. All constructs were subcloned into the EcoR I and Kpn I sites of the pEGFP-c1 vector (Clontech). The DNA sequence of each construct was confirmed by direct sequencing (GenScript.com).

### 2.2. Cell Culture and Transfection

HeLa cells were cultured in DMEM (Sigma-Aldrich, Saint Louis, MO, USA) containing 10% FBS (Invitrogen, Thermo Fisher Scientific, Karlsruhe, Germany) at 37 °C in a 5% CO_2_ atmosphere. An initial density of 1.5 × 10^5^ cells was seeded on glass coverslips incubated at 37 °C for 24 h, and then transfected with 2 μg of the designated plasmids using FuGene HD^®^ Transfection Reagent (Promega, Madison, WI, USA), according to the manufacturer’s instructions. Twenty-four or 48 h after transfection, cells were fixed with 4% paraformaldehyde (PFA) at room temperature for 15 min and washed with PBS at room temperature. Thereafter, cells were permeabilized with PBS supplemented with 0.2% Triton X-100 for 3 min, blocked with PBS containing 10% fetal bovine serum and 0.2% Tween 20 for 30 min, and then processed for immunohistochemistry.

### 2.3. HGPS and Normal Fibroblast Cultures

Fibroblasts from patients with HGPS were obtained from The Progeria Research Foundation Cell and Tissue Bank (http://www.progeriaresearch.org). The following fibroblasts were used: HGADFN003, HGADFN127, HGADFN155, and HGADFN164. Control fibroblasts were obtained from the Coriell Institute for Medical Research (Camden, NJ, USA). The following cell lines were used: GM01651C, GM03349C, and GM08398A. Cells were cultured using previously described methods [22].

### 2.4. Immunohistochemistry

After fixation, cells were subjected to indirect immunofluorescence staining with the following primary antibodies: goat-anti-lamin B1 (Santa Cruz Biotechnology, Heidelberg, Germany, M-20, sc-6217, 1:50), rabbit-anti-lamin C (Abcam, Cambridge, UK, ab125679, 1:500), mouse-anti-emerin (Leica Biosystems, NCL-Emerin, 4G5, 1:500), mouse-anti-NPC 414 (BioLegend, MMS-120P, Mab414, 1:2000), rabbit-anti-SUN1 (Sigma-Aldrich, HPA008346, 1:600), rabbit-anti-LC3B (Cell Signaling Technology #2775, 1:400), mouse-anti-LAMP-2 (Santa Cruz Biotechnology, sc-18822, 1:400), rabbit-anti-HP1β (Sigma-Aldrich, H2039,1:400), goat-anti-Ki67 (BD, 610968, 1:400), mouse-anti-EGFP (Clontech, 1:1000), rabbit-anti-progerin (*Monoclonal antibody* [30], 0.1 µg/mL), mouse-anti-progerin (Enzo, ALX-804-662, 13A4, 1:600), mouse-anti-Calnexin (Abcam, ab31290, 1:500), and mouse-anti-GM130 (BD, 610823, 1:100). The secondary antibodies used in the present study were affinity-purified Alexa Fluor^®^ 555 or 488 conjugated anti-goat/rabbit/mouse antibodies (Life Technologies, Carlsbad, CA, USA, A21432 anti-goat-555, A21206 anti-rabbit-488, A21202 anti-mouse-488, A31570 anti-mouse-555, and A31572 anti-rabbit-555, 1:1000). Cells were incubated with indicated primary antibodies for 1 to 2 h and washed with PBS containing 0.2% Tween 20 three times for 5 min each. Next, cells were incubated with the corresponding secondary antibodies for 1 h and washed with PBS three times for 5 min each. All samples were counterstained with DAPI in Vectashield mounting medium (Vector Inc., VEC-H-1200). Images were acquired using an Axio Imager D2 fluorescence microscope (Objective X63 oil objective, Carl Zeiss, Oberkochen, Germany) and a Zeiss AxioCam MRm or a Zeiss LSM 510 META confocal laser-scanning microscope (Carl Zeiss). 

### 2.5. Western Blot Analysis

Cell pellets were lysed in Laemmli Sample Buffer containing 5% β-mercaptoethanol (Bio-Rad), 1× protease inhibitor cocktail (Calbiochem, Darmstadt, Germany), and 10 mM PMSF. Protein samples were heated three times 2 min at 95 °C and vigorously vortexed between incubations to completely dissolve the extracts. Total protein concentrations were determined by dot plotting. The samples were electrophoretically resolved on 4–20% Mini-Protean^®^ TGX™ gel (Bio-Rad, Berkeley, CA, USA) and subsequently transferred onto nitrocellulose membranes (Amersham, NJ, USA) for antibody detection. The blot was incubated with a monoclonal mouse-anti-EGFP antibody (Clonetech, Fremont, CA, USA, 632569, 1:2000) overnight at 4 °C to determine the expression of EGFP fusion proteins. After three washes with PBS-T buffer containing 0.2% Tween 20 for 5 min each, the membrane was incubated with the corresponding horseradish peroxidase-conjugated secondary antibody (Jackson Immuno-Research Laboratories, West Grove, PA, USA). Protein bands were visualized using the enhanced Clarity Western ECL substrate (Bio-Rad). Chemiluminescent signals were captured using a ChemiDOC MP system (Bio-Rad).

### 2.6. Monodansylcadaverine (MDC) Staining Assay

The autophagic vacuoles and dead fibroblasts and transfected HeLa cells were determined using an Autophagy/Cytotoxicity Dual Staining Kit (Cayman Chemical Company, Ann Arbor, MI, USA). Cells were plated on glass coverslips and then probed with 1:1000 dilution of MDC in staining buffer (Cell-Based Assay Buffer Tablet dissolved in water). After 10 min incubation at 37 °C, cells were washed twice with assay buffer in the dark. Fluorescence microscopy was performed immediately after MDC staining. Autophagic vacuoles were detected using the filter set for DAPI detection. Propidium iodide (PI) staining was detected using the Cy3 Red filter set.

### 2.7. Statistical Analysis

For calculations of the percentage of Ki67 positive, 960 cells transfected with the EGFP vector alone, 919 EGFP-NLS-59-R-CSIM expressing cells, and 934 EGFP-NLS-PG-9-CSIM expressing cells were counted. Three independent immunohistochemistry experiments and counts were performed. 

The measurement of MDC fluorescence intensity was performed using ImageJ software. Cells of interest were selected using free form selection tools. The analysis was set to Area, Integrated Density, and Mean Gray Value. The total fluorescence of the cell was measured and a neighboring region without fluorescence was selected and measured as background reading. The corrected total cell fluorescence (CTCF) was calculated using the formula: CTCF = integrated density − (area of selected cell × mean fluorescence of background readings). The CTCF of 253 control cells and 244 HGPS fibroblasts was measured. 

For cell death evaluations, the percentages of PI-positive control and HGPS fibroblasts were evaluated. A total number of 317 cells for control and 309 HGPS fibroblasts were counted. Three independent experiments and counts were performed. All statistical analyses were performed using Student’s *t*-test. Two-tailed *p*-values were calculated and *p* < 0.05 was considered as significant. 

## 3. Results

### 3.1. Intracellular Dynamics of the Prelamin A and Progerin Carboxy-Terminal Fusion Proteins

**Figure 1:**
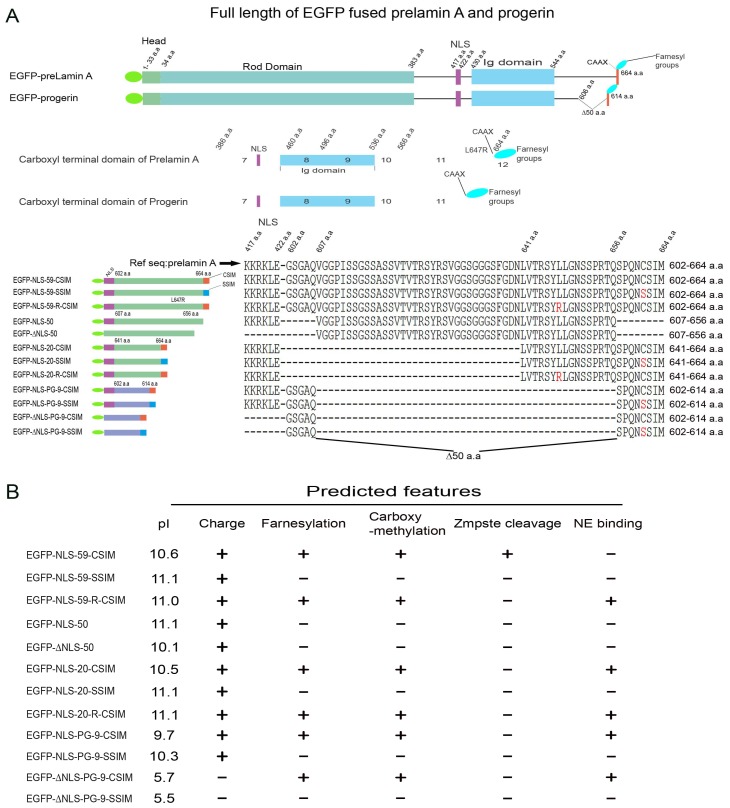
Schematic representation of the C-terminal fragments of prelamin A and progerin. (**A**) Schematics depicting the structures of the EGFP–prelamin A and EGFP–progerin proteins, and constructs derived from their C-terminal domains. The C-terminal domain of the indicated protein is enlarged and the alignment of the construct sequences is shown. (1) Residues from 602 to 664 a.a. (amino acids) are marked as 59-CSIM/SSIM/R-CSIM, which represents the wild type, C661S mutant, and L647R mutant C-terminal fragments of prelamin A, respectively; (2) The 50 a.a. construct encodes the 50 a.a. in-frame deletion of prelamin A from 607 to 656 a.a.; (3) Residues from 641 to 664 a.a. of prelamin A are marked as 20-CSIM/SSIM/R-CSIM, which represent the short form of wild type, C661S mutant, and L647R mutant C-terminal fragments of prelamin A, respectively; (4) The PG-9-CSIM/SSIM, residues from 602–614 a.a. of progerin, represents the C-terminal fragment of progerin and its corresponding C661S mutant form. A nuclear localization signal (NLS, KKRKLE) was linked to a part of the constructs as indicated; (**B**) Based on the protein sequence properties, predicted features of all constructs were indicated: pI value, static charge, and post-translational properties, including farnesylation, carboxymethylation, Zmpste cleavage, and nuclear membrane binding ability.

We generated a series of EGFP constructs encoding various sizes of the carboxy-terminal (CT) domains of prelamin A and progerin—including an NLS sequence and/or CaaX motif, as outlined in Figure 1A—to characterize the differences in the function of the CT domains of prelamin A and progerin. The sequences of the EGFP fusion proteins are indicated and their predicted post-translational modifications and NE interaction are also shown (Figure 1B). 

Western blot analyses showed that all EGFP fusion proteins were expressed in transfected HeLa cells at the expected sizes (Figure S1). NLS-59-CSIM (602–664 a.a.), corresponding to the CT domain of prelamin A, showed a slightly smaller size than NLS-59-R-CSIM (containing the L647R mutation) and NLS-59-SSIM (containing the C661S mutation), indicating that this protein underwent post-translational processing (Figure S1). NLS-50 (607–656 a.a.), which corresponds to the 50 a.a. that are missing in the progerin protein, was of similar size as NLS-59-CSIM. Thus, the NLS-59-CSIM protein was processed in a similar manner as full-length prelamin A, resulting in the production of a CT sequence similar to the corresponding mature lamin A CT domain [12]. The NLS-59-R-CSIM and NLS-59-SSIM proteins, however, were not cleaved and therefore corresponded to the CT end of prelamin A. 

The Zmpste 24 enzyme requires the last 41 a.a of the prelamin A CT for its efficient cleavage activity [31]. The NLS-20-CSIM and NLS-20-R-CSIM proteins were similarly sized but were slightly smaller than NLS-20-SSIM (Figure S1). Based on this result, both NLS-20-CSIM and NLS-20-R-CSIM were farnesylated but Zmpste 24 did not further process NLS-20-CSIM, probably due to the short a.a. sequence upstream of the Zmpste 24 cleavage site (Figure S1). The NLS-20-CSIM corresponds to the wild-type 20 a.a.–CaaX CT domain of prelamin A that maintained the last 15 amino acids and the farnesylated and carboxymethylated cysteine. The NLS-PG-9-CSIM, corresponding to the last 13 a.a. of the progerin CT domain, was slightly smaller in size than its counterpart, NLS-PG-9-SSIM, that cannot be farnesylated (Figure S1). Collectively, all EGFP fusion proteins were efficiently expressed in transfected HeLa cells, migrated at the expected molecular weights, and showed a certain degree of post-translational modifications (i.e., farnesylation and/or Zmpste 24 cleavage), according to the Western blot analyses. 

### 3.2. The Farnesylated CT Domains of Prelamin A and Progerin Are Sufficient for Inducing NE Defects

We examined the cellular localization of the various EGFP fusion proteins in transfected HeLa cells relative to lamin B1 using immunohistochemistry to identify the potential alterations in the subcellular targeting of the CT fragments of prelamin A and progerin.

**Figure 2:**
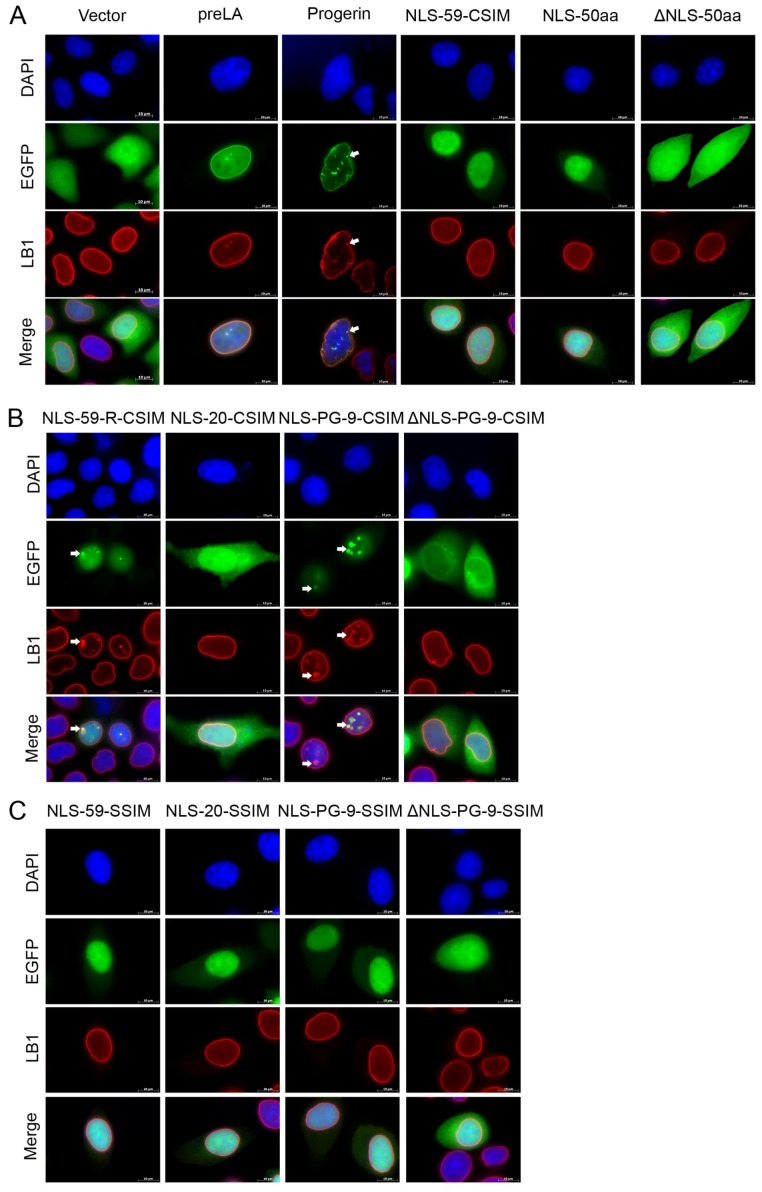
Determination of the cellular localization of EGFP plasmids in transiently transfected HeLa cells. Immunocytochemistry was performed on HeLa cells transfected with EGFP fusion proteins. Cells were stained with an anti-lamin B1 antibody. Chromatin was stained with DAPI. Representative images of lamin B1 staining in the indicated cells. (**A**) NLS directs the protein to the cell nucleus; (**B**) The farnesylated CaaX motif, along with the NLS, targets the EGFP–NLS-59-R-CSIM and EGFP–NLS-PG-9-CSIM proteins to the nuclear envelope (NE) and EGFP–NLS-20-CSIM protein to the NE and cytoplasm and membrane; (**C**) Proteins carrying the C661S mutation within the CaaX motif exhibit an intra-nucleoplasm diffusion pattern. Scale bar, 10 µm.

In HeLa cells transfected with the EGFP–vector, the EGFP signal spread throughout the cytoplasm and the nucleus, and typical nuclear lamin B1 rim-like staining was observed (Figure 2A). The EGFP–prelamin A fusion protein showed a nuclear rim signal overlapping lamin B1 staining at the NE (Figure 2A). The EGFP–progerin signal colocalized with lamin B1 at the NE and NE invaginations, with most of the transfected cells showing an abnormal nuclear morphology (Figure 2A). The EGFP–NLS-59-CSIM and EGFP–NLS-50 proteins were distributed in a diffused pattern in the nuclear compartment (Figure 2A). This same nuclear localization of EGFP–NLS-59-CSIM and EGFP–NLS-50 signified that NLS-59-CSIM underwent the same complete post-translational modifications as prelamin A and was no longer farnesylated. The EGFP–ΔNLS-50aa protein lacking the NLS exhibited a diffuse distribution throughout the cytoplasm and nucleus of transfected cells (Figure 2A). 

The EGFP–NLS-59-R-CSIM signal was colocalized with lamin B1 at the NE (Figure 2B). Abnormal nuclear shape was observed and enlarged NE structures emanating from the NE were positive for the EGFP signal (Figure 2B and Figure S2). Lamin B1 and EGFP–NLS-59-R-CSIM signals were superimposable at the NE evaginations (Figure 2B and Figure S2). The EGFP–NLS-20-CSIM protein was targeted to the NE, and a fraction of the protein was also detected at the plasma membrane and in the cytoplasmic compartments (Figure 2B). Further immunodetection of GM130, a protein marker of the Golgi apparatus, showed that the EGFP–NLS-20-CSIM signal accumulated in the Golgi apparatus (Figure S3A) [32]. According to a previous report, the hypervariable region (HVR) of RAS proteins undergoes multiple post-translational modifications including CAAX farnesylation, carboxy-terminal methylation, and palmitoylation. The processed RAS proteins traffic through the classical secretory pathway via the Golgi to the plasma membrane, with the exception of K-Ras4B, which bypasses the Golgi [33,34]. Based on this observation, we compared the protein sequences of NLS-20-CSIM with the HVRs of H-Ras, N-Ras, K-Ras4A, and K-Ras4B (Figure S3B). This sequence alignment and similarity analysis indicated a certain degree of similarity between the NLS-20-CSIM and the HVRs of RAS proteins (Figure S3B). Thus, NLS-20-CSIM might be trafficked from Golgi to the plasma membrane via the same route as RAS proteins. The EGFP–NLS-PG-9-CSIM protein localized with lamin B1 at the NE and within NE evaginations, as observed in EGFP–NLS-59-R-CSIM-transfected cells (Figure 2B and Figure S2). The EGFP–ΔNLS-PG-9-CSIM protein, however, was localized in the cytoplasm and at sites around the NE on the cytoplasmic face of the NE (Figure 2B). Based on these results, EGFP fusion proteins that contained a terminal CSIM underwent farnesylation and fragments that remained farnesylated localized at the NE, with the exception of NLS-20-CSIM, which was also present to a lesser extent at the cytoplasmic membrane. 

We generated mutants of these proteins by modifying the cysteine at position 661 to a serine residue (C661S), thus changing the CSIM to SSIM that is no longer farnesylated, to confirm that the farnesylation of the cysteine residue within the CSIM is required for the membrane interaction and NE localization (Figure 2C). These EGFP fusion proteins ending with a SSIM and containing an NLS sequence were not detected at the NE but were localized throughout the nucleoplasm, and regular lamin B1 rim staining was observed (Figure 2C). Moreover, EGFP–ΔNLS-PG-9-SSIM did not accumulate around the NE on the cytoplasmic face, indicating that the EGFP–ΔNLS-PG-9-CSIM fragment was farnesylated and localized to the outer NE (Figure 2B,C). Further analysis of EGFP–ΔNLS-PG-9-CSIM distribution in relation to GM130, a Golgi marker [32], and calnexin, a marker of the endoplasmic reticulum [35], showed that populations of this short farnesylated progerin peptide were localized in both compartments (Figure S3A).

Therefore, the farnesyl modification is responsible for targeting the EGFP fusion proteins to the NE and the induction of NE abnormalities and nuclear shape alterations, as observed for the full-length progerin protein in transfected HeLa cells or HGPS fibroblasts [36]

### 3.3. NE Proteins Are Mislocalized by the Farnesylated CT of Prelamin A and Progerin

Because the EGFP–NLS-59-R-CSIM and EGFP–NLS-PG-9-CSIM proteins induced NE deformations and evagination, we sought to evaluate their impacts on nuclear lamin B1, lamin C, and NE proteins—including emerin, nuclear pore complexes (NPC), and SUN1—in transfected HeLa cells. 

**Figure 3:**
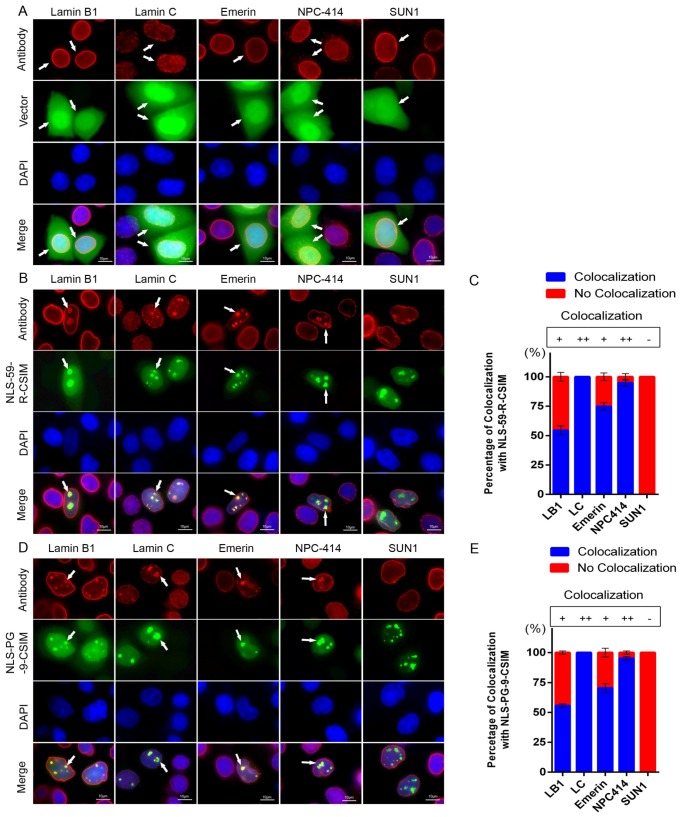
Disorganized distributions of NE proteins. (**A**) Immunocytochemistry was performed on EGFP vector-, (**B**) EGFP–NLS-59-R-CSIM- and (**D**) EGFP–NLS-PG-9-CSIM-transfected HeLa cells. In panels **A**, **B**, and **D**, cells were stained with anti-lamin B1, lamin C, emerin, NPC-414, and SUN1 antibodies. Chromatin was stained with DAPI. Representative images of lamin B1, lamin C, emerin, NPC-414, and SUN1 staining in the indicated cells. Colocalization of lamin B1, lamin C, emerin, and NPC-414 with EGFP signals at the NE and NE invaginations are as indicated with arrowheads. Scale bar, 10 µm. (**C**,**E**) Percentages of cells displaying colocalization of the NE protein and EGFP signal in the images shown. More than 300 EGFP-positive cells for each construct were counted, *n* = 3.

Cells transfected with the empty EGFP vector exhibited a typical NE rim signal for lamin B1, lamin C, emerin, NPC-414, and SUN1 proteins (Figure 3A). In EGFP–NLS-59-R-CSIM-transfected cells, lamin B1, lamin C, emerin, and NPC-414 were all colocalized with the EGFP signal at the NE and the NE evaginations (Figure 3B, arrowheads). Meanwhile, SUN1 was present at the NE, but not in NE evaginations (Figure 3B). Notably, 54.33 ± 3.81% of lamin B1, 100% of lamin C, 74.78 ± 3.27% of emerin, and 94.86 ± 2.69 of NPC-414 signals overlapped with EGFP–NLS-59-R-CSIM at the NE invaginations (Figure 3C). In contrast, a barely detectable SUN1 signal was observed at the NE tubular structures (Figure 3C).

A similar distribution pattern was also observed for EGFP–NLS-PG-9-CSIM and lamin B1, lamin C, emerin, and NPC-414 at the NE and NE protrusions (Figure 3D, arrowheads), whereas SUN1 was also absent from the NE evaginations in cells transfected with this construct. Colocalization frequencies indicated that EGFP–NLS-PG-9-CSIM overlapped with 55.72 ± 1.38% of lamin B1, 100% of lamin C, 70.51 ± 3.64% of emerin, and 95.17 ± 1.39% of NPC-414 signals at the NE evaginations, and SUN1 was absent from these NE protrusions (Figure 3E). Furthermore, cells expressing high levels of both EGFP fusion protein fragments, particularly EGFP–NLS-20-CSIM, exhibited a higher degree of nuclear abnormalities (Figures S4–S6). Thus, the degree of NE disorganization directly depended on the amount of these protein fragments expressed in transfected HeLa cells. In contrast, this short fragment lacking the NLS (EGFP–ΔNLS-PG-9-CSIM) accumulated in the Golgi apparatus, ER, and outer nuclear membrane, but had less of an impact on the NE shape (Figure S3A). Based on these results, the farnesylated CT fragments of prelamin A and progerin induced NE deformation by interfering with the distribution of lamin B1, lamin C, and the inner NE proteins, emerin and NPC. 

### 3.4. NE Deformation Induces Heterochromatin Disorganization and Reduces Cell Proliferation

**Figure 4:**
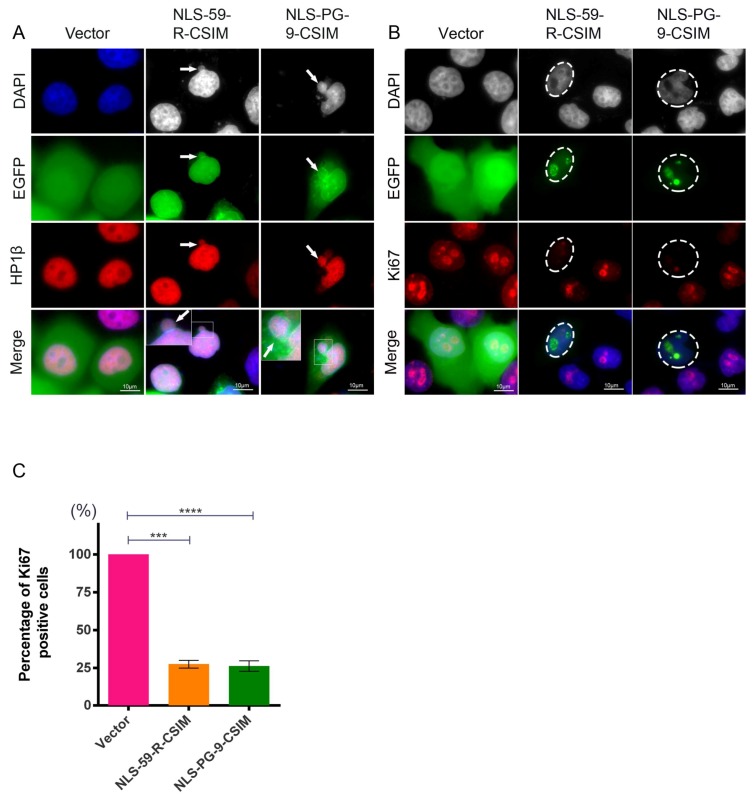
Determination of the heterochromatin organization and cell proliferation activity. (**A**) Heterochromatin was stained with an anti-HP1β antibody. Representative images of HP1β staining in EGFP vector-, EGFP–NLS-59-R-CSIM-, and EGFP–NLS-PG-9-CSIM-transfected cells. Cytoplasmic HP1β staining shows the disorganized heterochromatin signals in EGFP–NLS-59-R-CSIM- and EGFP–NLS-PG-9-CSIM-transfected cells, as indicated by the arrowheads. Higher magnification images show the colocalization of DAPI and heterochromatin staining in the cytoplasm. Scale bar, 10 µm; (**B**) Ki67 was used to detect the actively replicating chromosomal DNA. Representative images of Ki67 staining and signals for EGFP fusion proteins are shown. Ki67 signals are barely detectable in EGFP–NLS-59-R-CSIM- and NLS-PG-9-CSIM-expressing cells, as indicated with circles; (**C**) Percentages of cells expressing both EGFP and Ki67. Nine hundred sixty cells expressing the EGFP vector, 919 cells expressing EGFP–NLS-59-R-CSIM, and 934 cells expressing EGFP–NLS-PG-9-CSIM were counted (*n* = 3). Scale bar, 10 µm.

Because lamin B1 and emerin are intimately linked to heterochromatin organization, we evaluated the distribution of the heterochromatin protein HP1β [37]. In cells transfected with the EGFP vector alone, HP1β was exclusively located within the nucleoplasm (Figure 4A). However, in both EGFP–NLS-59-R-CSIM- and EGFP–NLS-PG-9-CSIM-expressing cells, the HP1β signal was also detected in some cytoplasmic areas emanating from the NE (Figure 4A, arrowheads). Moreover, the HP1β signal in these blebs colocalized with DAPI staining, indicating that these foci contained DNA (Figure 4A). Therefore, both EGFP-farnesylated CT fragments induced the loss of chromatin from the nuclear compartment. 

We postulated that these chromatin aggregates in the cytoplasm might arise from altered mitotic events and examined the distribution of Ki67, a proliferation marker and a protein detected on actively replicating chromosomes [38]. Cells expressing the empty EGFP vector exhibited numerous large Ki67-positive speckles in the nucleus, indicating that the nuclei were replicating and, therefore, these cells were proliferating (Figure 4B). In contrast, the Ki67 signal was barely detectable in cells transfected with EGFP–NLS-59-R-CSIM and EGFP–NLS-PG-9-CSIM (Figure 4B, circled region), indicating that these cells were not replicating. Based on these findings, cells transfected with these CT-farnesylated fragments were not mitotically active. For the statistical analysis of three independent experiments, 919 EGFP–NLS-59-R-CSIM-positive cells and 934 EGFP–NLS-PG-9-CSIM-positive cells were counted, and the number of Ki67-positive cells among these populations was determined (Figure 4C). Only 27.5 ± 2.5% of EGFP–NLS-59-R-CSIM cells and 26.3 ± 3.5% NLS-PG-9-CSIM cells were Ki67-positive and therefore remained mitotically active (*p* < 0.001, Figure 4C). These numbers were significantly reduced compared to EGFP vector-expressing cells (Figure 4C). Thus, these two farnesylated CT fragments significantly reduced the growth rate of transfected cells, possibly by interfering with the chromatin distribution during mitosis, as indicated by the presence of DNA and HP1β in the nuclear and cytoplasmic blebs of transfected cells. 

### 3.5. The Autophagy–Lysosome Machinery Is Involved in the Formation of NE Evaginations

The observed NE defects and genomic instability in EGFP–NLS-59-R-CSIM- and EGFP–NLS-PG-9-CSIM-expressing cells indicated that several nuclear components were disrupted. The turnover of nuclear components is mediated by autophagy [39,40]. We determined the distribution of LC3B, a marker of autophagosomes [41], to determine whether components of the autophagy machinery were recruited to sites of NE blebs.

**Figure 5:**
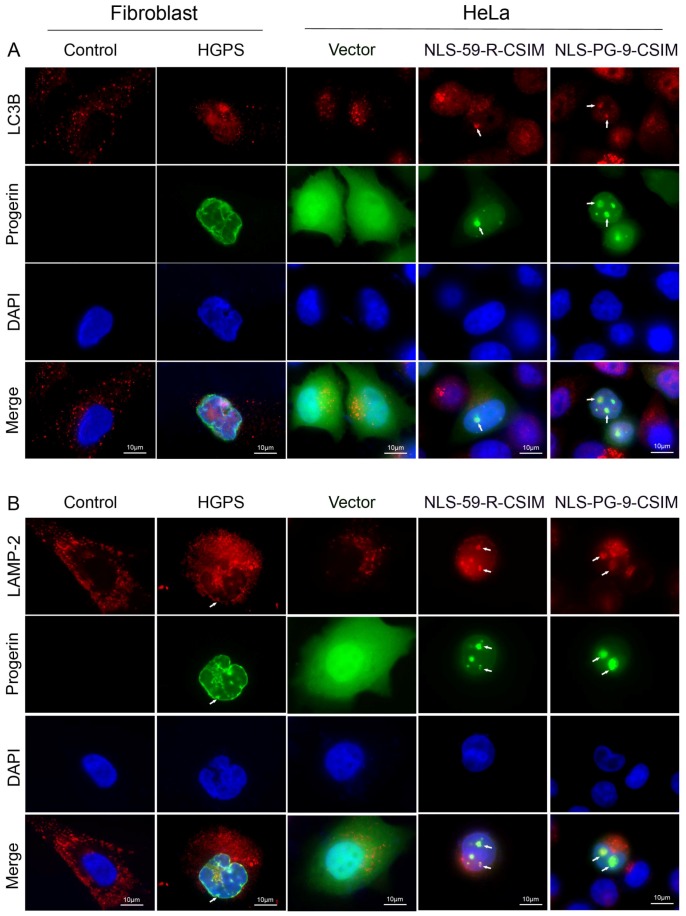
Detection of autophagosome and lysosome distributions. (**A**) LC3B and (**B**) LAMP-2 were used as markers to detect autophagosomes and lysosomes, respectively. Chromatin was stained with DAPI. Representative images of LC3B and LAMP-2 staining in normal and Hutchinson–Gilford progeria syndrome (HGPS) fibroblasts and EGFP vector-, EGFP–NLS-59-R-CSIM-, and EGFP–NLS-PG-9-CSIM-transfected cells. Redistribution and colocalization patterns of LC3B and LAMP-2 in the NE and NE invaginations are shown in HGPS fibroblasts and EGFP–NLS-59-R-CSIM- and EGFP–NLS-PG-9-CSIM-transfected cells, as indicated with arrowheads. Scale bar, 10 µm.

First, we analyzed the distribution of LC3B in primary fibroblasts derived from normal individuals and patients with HGPS. In control fibroblast cells, the punctate form of the LC3B signal was spread throughout the cytoplasm (Figure 5A, Figure S8). In HGPS fibroblast cells, the cytoplasmic LC3B signal was reduced, but a fraction of LC3B localized at the nuclear compartment, indicating that autophagosomes were located in the vicinity of the deformed NE (Figure 5A, arrowheads). In HeLa cells transfected with empty vector, LC3B foci were detected in the cytoplasm (Figure 5A). In HeLa cells transfected with EGFP–NLS-59-R-CSIM and EGFP–NLS-PG-9-CSIM, the EGFP signal was colocalized with LC3B-positive vesicles at the nuclear compartment (Figure 5A). EGFP–LC3B-positive foci were not observed at the NE of non-transfected cells. 

Next, we examined the localization of LAMP-2, a lysosomal membrane marker [42]. In control fibroblasts, the vacuolar signal of LAMP-2 was spread throughout the cytoplasm (Figure 5B and Figure S8). In HGPS fibroblast cells, in addition to the cytoplasmic signal, a granular staining pattern of LAMP-2 also surrounded the nucleus (Figure 5B). A fraction of LAMP-2 colocalized with progerin at the periphery of the NE fold in HGPS cells (Figure 5B, arrowheads). In HeLa cells transfected with the vector alone, the LAMP-2 signal was distributed in the cytoplasm, as observed in normal fibroblasts (Figure 5B). In EGFP–NLS-59-R-CSIM- and EGFP–NLS-PG-9-CSIM-expressing cells, LAMP-2 and EGFP were colocalized at the NE periphery and NE evaginations, as observed in HGPS fibroblast (Figure 5B). Therefore, LC3B and LAMP-2 were recruited to the NE in cells expressing EGFP–CT farnesylated fragments of prelamin A or progerin. Based on these observations, components of the autophagy–lysosome system are recruited to the nuclear periphery of HGPS cells and HeLa cells expressing the farnesylated C-terminal ends of prelamin A or progerin. The expression of these short farnesylated proteins induced NE evaginations and LC3B recruitment. LAMP-2 was also detected in the vicinity of the NE evaginations in HeLa cells transfected with these constructs. Thus, the accumulation of farnesylated proteins in the nuclear compartment activates nuclear autophagy to possibly maintain the integrity of the nucleus.

### 3.6. NE Defects Are Associated with Lower Autophagy Activity and Increased Cell Death

**Figure 6:**
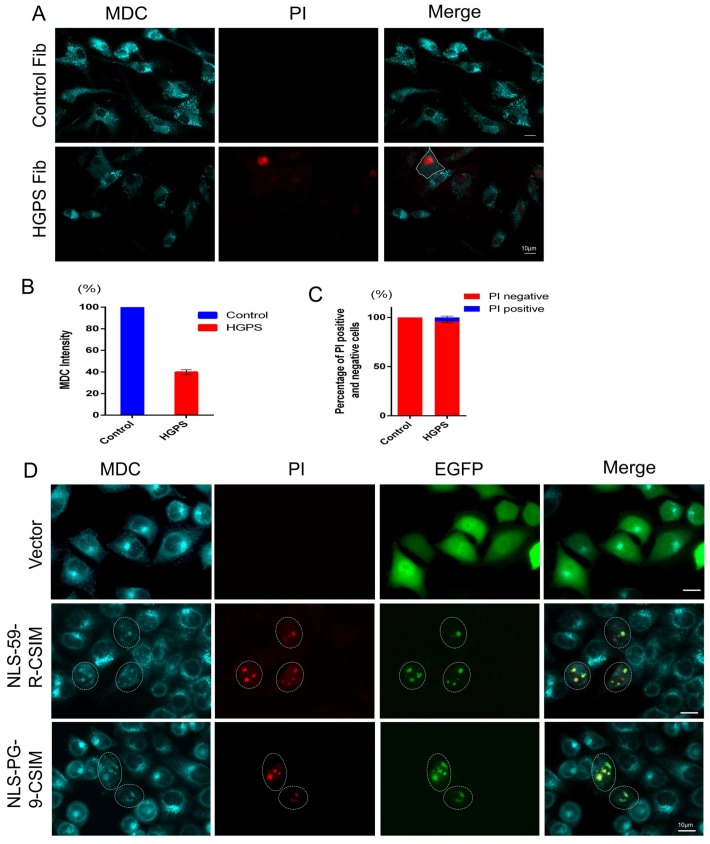
Determination of autophagy activity. (**A**) Monodansylcadaverine (MDC) and propidium iodide (PI) staining were performed to detect the numbers of autophagosomes and dead cells in normal and HGPS fibroblasts. Representative images of cells are shown. HGPS cells exhibit heterogeneous brightness of MDC fluorescence intensity. A PI-positive cell with no detectable MDC signal is outlined; (**B**) The fluorescence intensity of MDC was measured in control and HGPS fibroblasts. Two hundred fifty-three control cells and 244 HGPS cells were counted. The average MDC fluorescence intensity was used for the statistical analysis; (**C**) The percentages of PI-positive cells were calculated by counting 317 control cells and 309 HGPS fibroblasts. All statistical analyses were performed using Student’s *t*-test. Two-tailed *p*-values were calculated and *p* < 0.05 was considered significant; (**D**) MDC and PI staining in EGFP vector-, EGFP–NLS-59-R-CSIM-, and EGFP–NLS-PG-9-CSIM-transfected cells. EGFP–NLS-59-R-CSIM- and EGFP–NLS-PG-9-CSIM-transfected cells showing colocalization of MDC with the EGFP signal at the NE invaginations are circled. Scale bar, 10 µm.

HGPS cells display reduced autophagy activity [36,43]. Because components of autophagosomes were recruited to the NE invagination in HGPS cells and HeLa cells expressing EGFP–CT farnesylated fragments, we evaluated autophagy activity in these cells. We applied a monodansylcadaverine (MDC)/cytotoxicity dual staining assay in living cells that enables the simultaneous detection of autophagy and cell death. MDC is a fluorescent probe that detects autophagic vacuoles in living cells and propidium iodide (PI) is a marker of cell death [44]. A bright vacuolar signal of autophagosomes (MDC) distributed throughout the cytoplasm was observed in control cells, and no PI-positive cells were detected, indicating the absence of cell death (Figure 6A,C). In contrast, HGPS fibroblasts exhibited an average decrease in the MDC signal of 60% compared to control fibroblasts, signifying a reduction in the number of autophagosomes (Figure 6A,B). In addition, an increase in the percentage of dead cells of 4.22 ± 1.27% was observed in HGPS cells compared to control fibroblasts (Figure 6C). Hence, in PI-positive HGPS, the MDC signal was barely detectable (Figure 6A). Collectively, these results are consistent with previous findings that autophagy is reduced in HGPS fibroblasts [36,45,46]. 

All HeLa cells transfected with the empty EGFP vector showed a strong MDC signal and no PI-positive signals, indicating the absence of dead cells (Figure 6D). In EGFP–NLS-59-R-CSIM- and EGFP–NLS-PG-9-CSIM-expressing HeLa cells, the cytoplasmic signal for MDC was dramatically reduced (Figure 6D, circled region). However, superimposable bright signals for EGFP, MDC, and PI were detected at the NE evaginations in transfected HeLa cells (Figure 6D). The presence of the PI signal within these NE blebs indicates that the NE permeability was compromised, and cells were undergoing apoptosis. 

Based on these findings, the accumulation of farnesylated proteins at the NE induces NE alterations and the disorganization of the numerous nuclear components, including NE proteins (emerin and SUN1), lamins, chromatin, and other nuclear factors. These changes apparently induce the formation of NE evaginations, the recruitment of autophagy components, and the activation of nucleophagy in an attempt to prevent further nuclear alterations. However, the dramatic disruption of the NE integrity ultimately leads to apoptosis. 

## 4. Discussion

At least 15 inherited diseases called laminopathies are linked to *LMNA* mutations that cause the characteristic abnormal nuclear morphology [47,48]. Currently, progerin accumulation at the NE is known to produce dysmorphic nuclei in patients with HGPS [49]. Likewise, a loss of Zmpste 24 activity in progeroid mice causes the accumulation of farnesylated prelamin A at the NE, which also induces NE abnormalities [50]. Further investigations are required to determine the mechanisms by which farnesylated progerin and prelamin A interact with NE proteins and other components to induce nuclear structural abnormalities. The present study provides new evidence that the farnesylated carboxy-terminal moieties of prelamin A and progerin play critical roles in NE association and deformation. Hence, this study highlights an important role of the autophagy–lysosome system in the maintenance of the integrity of the NE and nuclear structure.

### 4.1. Subcellular Trafficking of the Farnesylated Progerin Carboxyl-Terminal Fragment

We dissected the structure and function of the progerin CT domain to determine the mechanism by which modifications to the progerin carboxy-terminus cause NE defects in HGPS cells. We created a series of plasmids that encode the progerin CT domain and the wild-type preLA CT domain, as outlined in Figure 1A. We analyzed the distribution of the EGFP fusion proteins with or without a nuclear localization signal (NLS) and a functional CAAX motif to provide novel insights into progerin processing and intracellular trafficking in transfected HeLa cells. Progerin farnesylated CT fragments with an NLS motif induced NE deformation in transfected HeLa cells and fibroblasts from patients with HGPS, similar to the full-length progerin protein [21,36]. The farnesylated CT–preLA and –progerin fragments were mainly localized at the NE. Nuclear accumulation of these prenylated fusion proteins induced NE deformation, including blebs and NE elongations or protrusions of various sizes and numbers. These NE protrusions were not observed in cells transfected with the full-length progerin cDNA or in HGPS fibroblasts, which showed NE invaginations and small blebs, as previously reported [36]. These NE protrusions were not observed in HeLa cells expressing non-farnesylated CT–preLA or –progerin fusion proteins, which were localized throughout the nucleoplasm. Hence, EGFP–NL-59-CSIM and EGFP–NLS-50 proteins corresponding to the 50-amino acid fragment that is missing in the progerin protein showed a diffuse distribution in the nucleus, indicating that this protein fragment does not associate with any particular subnuclear compartment. Meanwhile, the EGFP–NLS-59-R-CSIM protein corresponding to the farnesylated 50 amino acid fragment was localized at the NE, indicating that the CAAX modification promoted the membrane interaction, as previously reported [51,52]. Consistent with these observations, the farnesylated progerin CT peptide without the NLS (EGFP–ΔNLS-PG-9-CSIM) was localized at the ONM that is continuous with the ER compartment. Cells expressing high levels of EGFP–ΔNLS-PG-9-CSIM showed accumulation of this fragment in the Golgi apparatus. Based on this observation, the short farnesylated progerin CT peptide was attached to the ER membrane and transported to the Golgi compartment.

The subcellular distribution of the short NLS CT–preLA peptide (EGFP–NLS-20-CSIM) showed NE localization and accumulation in the Golgi apparatus and the plasma membrane, whereas the NLS CT–progerin peptide (EGFP–NLS-PG-9-CSIM) was restricted the NE. We compared the amino acid sequence of these two short peptides and performed protein sequence alignments using Predict Protein software [53] to understand the differences in the localization of these peptides. In contrast to NLS-PG-9-CSIM, NLS-20-CSIM showed some degree of similarity with the hypervariable region (HVR) of the RAS proteins (H-RAS, N-RAS, K-RAS4A, and K-RAS4B), which are also known to contain a CAAX motif [33]. K-RAS is targeted from the ER to Golgi and the plasma membrane immediately after post-translational modification [33]. K-RAS contains a polybasic sequence that is comparable to the NLS sequence and is located upstream of its CAAX motif. Furthermore, the distance between the two motifs (NLS and CAAX) are critical for plasma membrane targeting, as EGFP–NLS-59-R-CSIM exhibited only nuclear localization at the NE [54]. According to our findings, the NLS-20-CSIM protein residing in the ER after prenylation by the specific enzymes was targeted to the nucleus or transported to the Golgi and the plasma membrane, as previously reported for K-RAS protein [34]. Moreover, high levels of farnesylated proteins tend to accumulate at the Golgi.

### 4.2. Farnesylated CT–Progerin and –preLA Peptides Dislodge SUN1 from NE Protrusions 

Previous studies, including studies from our group, have shown that progerin colocalizes with SUN1 in interphase HGPS fibroblasts [27,28,29]. Progerin accumulated at the NE and codistributed with SUN1 at the NE invaginations or blebs but did not induce NE elongations in interphase HGPS fibroblasts or transfected HeLa cells. As shown in our previous report, the spatiotemporal distribution of SUN1 during mitosis is substantially altered in the presence of progerin. SUN1 recruitment to the nuclear periphery is delayed in anaphase, and SUN1 predominately codistributes with progerin at the ER membrane structures in mitotic HGPS cells [29]. According to a study by Chen et al., SUN1 not only binds lamin A but also binds strongly to farnesylated progerin and preLA, indicating that the farnesyl moiety was partially responsible for the stronger association with SUN1 [27]. SUN1 is a component of the multifunctional nuclear membrane protein assembly called the linker of nucleoskeleton and cytoskeleton (LINC) complex, which consists of the INM-spanning protein SUN and the ONM-spanning protein nesprin [55]. The crystal structure of the LINC complex indicates that this assembly involves three KASH peptides interacting with a SUN trimer [56]. Additionally, a disulfide bond covalently links the SUN and KASH domains, thereby bridging the INM and the ONM and creating a force-resistant device that permits mechanical signal transmission across the NE [56,57]. Moreover, it has been suggested that SUN trimers within the perinuclear space could form higher-order clusters by lateral association of their SUN domains [58]. In the context of HGPS fibroblasts, because SUN1 is increased at the NE compared to normal fibroblasts, SUN1 clustering might be favored, thereby increasing the stiffness of the NE. Moreover, this LINC complex directly connects the cytoskeleton (e.g., actin filaments or microtubule motors) and the nucleoskeleton (e.g., lamins or chromatin) and plays a major role in shaping and positioning the nucleus and in cell migration [57,59]. HGPS cells with the most dysmorphic nuclei resulting from high levels of progerin accumulation exhibit reduced migration potency [21]. This observation suggests that increased LINC connections at the NE would disrupt the nucleoskeleton and cytoskeleton networks inhibiting cell migration. By contrast, a recent study indicates that reduced levels of SUN1 and other LINC-associated components in cancer cells cause a decrease in cellular rigidity and, consequently, increase cell migration [60].

In the present study, we further investigated the impact of the CT–progerin (EGFP–NLS-PG-9-CSIM) and CT–preLA (EGFP–NLS-59-R-CSIM) peptides on the distribution of SUN1. At the nondeformed area of the NE, the farnesylated protein fragments were colocalized with lamin A/C, lamin B1, and the INM proteins emerin and SUN1, as well as with the nuclear pores (NPCs). By contrast, within the large NE protrusions, the EGFP signal was colocalized with all of the abovementioned nuclear constituents, with the exception of SUN1. The NE protrusions were depleted of SUN1 but contained the INM protein emerin and the NPCs, indicating that these NE elongations are composed of the double-membrane bilayers of the INM and the ONM. SUN1 may have been excluded from sites of NE elongation due to the membrane flexibility required to allow NE outgrowth. Indeed, as part of the LINC complex, SUN1 forms immobile and rigid connections among the NE, the nuclear lamina, and the cytoskeleton [61]. The LINC complex requires a degree of disassembly to permit membrane flexibility and allow NE outgrowth, whereas SUN1 clustering might permit local NE flexibility to allow NE evaginations, as indicated in this study. SUN1 loosens its connection to the nuclear lamina during mitosis [62]. However, during nuclear breakdown, the LINC complex remains stable [62]. Whether SUN1 or the LINC complex can disassemble in interphase nuclei remains to be investigated. Therefore, based on our findings, macroprotein complexes containing SUN1 might restrict NE outgrowth, which otherwise would cause NE rupture, whereas SUN1 depletion would facilitate NE elongation. Further studies are needed to address this question in more detail and validate this hypothesis.

### 4.3. Autophagy Is Involved in the Formation of NE Protrusions

In the present study, a fraction of heterochromatin, as indicated by the DAPI and the HP1β signals, was present in cytoplasmic area close the nucleus in EGFP–NLS-59-R-CSIM- and EGFP–NLS-PG-9-CSIM-expressing cells. Thus, nuclear accumulation of those farnesylated protein fragments not only compromised the NE structure but also induced chromatin disorganization, which consequently caused genomic instability. Moreover, these transfected cells showed reduced Ki67 signals, indicating that they no longer proliferated. To understand why transfected cells with such atypical dysmorphic nuclei persisted in these cultures, we investigated whether these cells attempted to eliminate their damaged NE via autophagy to support their survival. Autophagy is a catabolic membrane trafficking process that degrades a variety of cellular constituent. The presence of key autophagy components within the nucleus, including microtubule-associated protein 1/light chain 3 (LC3B), has suggested a role for autophagy in the turnover of nuclear components [41]. Autophagy has recently been shown to target nuclei in mammalian cells [40,63,64].

We performed an immunohistochemical analysis of microtubule-associated protein 1/light chain 3 (LC3B) in HGPS fibroblasts and transfected HeLa cells to determine whether these elongated NE structures were autophagic vacuoles. LC3B is a commonly used marker of autophagy because it associates with the inner and outer membranes of autophagosomes [65].

LC3B immunostaining showed a predominant localization of the signal in the cytoplasm of normal fibroblasts, whereas in HGPS fibroblasts, the signal was concentrated at the nuclear compartment. HeLa cells transfected with EGFP–NLS-59-R-CSIM and EGFP–NLS-PG-9-CSIM showed extensive colocalization of LC3B with the EGFP signal at sites of NE elongations. We also examined the involvement of lysosomes to further characterize the autophagic nature of these NE protrusions, and found that LAMP-2, a lysosomal membrane protein, was present in the vicinity of the EGFP signal in HeLa cells transfected with EGFP–NLS-59-R-CSIM and EGFP–NLS-PG-9-CSIM. In HGPS cells, the LAMP-2 signal was also detected at the periphery of the NE. Collectively, these findings indicate that autophagosome/autolysosomes were present at the nuclear periphery in HGPS cells. However, transfected HeLa cells that had accumulated high amounts of farnesylated CT–progerin or –preLA fragments formed large NE protrusions that were labeled with markers of autophagosomes/autolysosomes. Thus, cells formed these structures in attempt to eliminate these toxic farnesylated EGFP-fusion proteins. Moreover, these elongated NE autophagosomes also contained lamina components (lamin A/C and lamin B), the INM protein emerin, chromatin, and chromatin-interacting proteins (HP1B), as well as NPCs. However, SUN1 was not detected in these NE autophagosome-like structures, suggesting that SUN1 might either be degraded by another mechanism and/or be excluded from these structures because of its inherent function in maintaining the shape and rigidity of the NE through its role in the LINC complex formation [55]. 

Remarkably, elongated NE autophagosomes were not observed in HGPS fibroblasts expressing high levels of progerin. The lack of these giant protrusion in HGPS cells might be related to the tight association of progerin with the nuclear lamina and particularly with SUN1, as reported previously [27]. Strong progerin interactions with the lamina meshwork and the INM might reduce progerin mobility at the NE in interphase nuclei and consequently reduce its rate of degradation via autophagy. Thus, progerin degradation might occur at the end of mitosis when a large amount of progerin remains trapped with SUN1 in the cytoplasm, although further investigations are needed to validate this assumption [27,29]. Nevertheless, based on accumulating evidence, progerin is degraded via autophagy, and the activation of the autophagy pathway in HGPS cells by various drugs, including rapamycin and sulforaphane, significantly enhances progerin clearance and ameliorates the HGPS cellular phenotype [36,43,46,66,67,68]. Studies aiming to improve our understanding of nucleophagy-dependent degradation of progerin in HGPS cells are needed to develop novel therapeutics to further modulate nuclear autophagy in patients with HGPS and possibly other conditions. 

This study also provides a plausible answer to the question of what becomes of the farnesylated 15-amino acid fragment cleaved from the preLA after normal processing. In a previous study by Sinenski et al., the cleaved CT farnesylated preLA peptide was suggested to play a role as signal peptide [69]. Numerous studies have investigated the processing of preLA; however, quite surprisingly, little is known about the outcome of the cleaved C-terminal 15-amino acid farnesylated moiety [12,70,71]. In the current study, the CT farnesylated progerin fragment (NLS-PG-9-CSIM) associated with the NE and was targeted for degradation via nucleophagy. Analogously to the progerin–CT peptide, the cleaved CT–preLA peptide most likely follows a similar degradation route. Therefore, this CT–preLA peptide probably has no additional functions other than targeting preLA to the NE, where it is modified to produce mature lamin A. Based on our findings, a better understanding of the mechanisms regulating the degradation of farnesylated lamins (progerin, prelamin A, and lamin B) is needed, and future studies should illuminate mechanisms to enhance the clearance of these proteins and restore the phenotype of progeria cells. 

## Supplementary Materials

The following are available online. Figure S1: Western Blot analyses of EGFP-fusion proteins expressed in transiently transfected HeLa cells, Figure S2: Abnormal nuclear envelope invaginations, Figure S3: Trafficking of EGFP-NLS-20-CSIM and distribution of EGFP-ΔNLS-PG-9-CSIM, Figure S4: Concentration-dependent NE protein disorganization in EGFP-NLS-59-R-CSIM-expressing cells, Figure S5: Concentration-dependent nuclear shape abnormalities in EGFP-NLS-20-CSIM-expressing cells, Figure S6: Concentration-dependent NE protein disorganization in EGFP-NLS-PG-9-CSIM-expressing cells, Figure S7: Immunodetection of NE proteins, cytoplasmic localization, and autophagosome-lysosome interactions in ∆NLS-PG-9-CSIM-expressing cells, Figure S8: Autophagosome and lysosome distributions.
