# Supplementary material for: Autophagic Removal of Farnesylated Carboxy-Terminal Lamin Peptides
Source: Cells. 2018 Apr 23;7(4):33. doi: 10.3390/cells7040033 (PMC5946110; doi:10.3390/cells7040033)
Supplement: Supplementary file 1 [file cells-07-00033-s001.zip › cells-282318-SI.pdf]

Supplementary Materials for

Autophagic removal of farnesylated carboxy-terminal lamin peptides

Xiang Lu [1] and Karima Djabali [1,*]

[1] Epigentics of Aging, Department of Dermatology, TUM School of Medicine, Technical University of Munich, 85748 Garching-Munich, Germany

* Correspondence: djabali@tum.de; Tel.: +49-89-289-10920

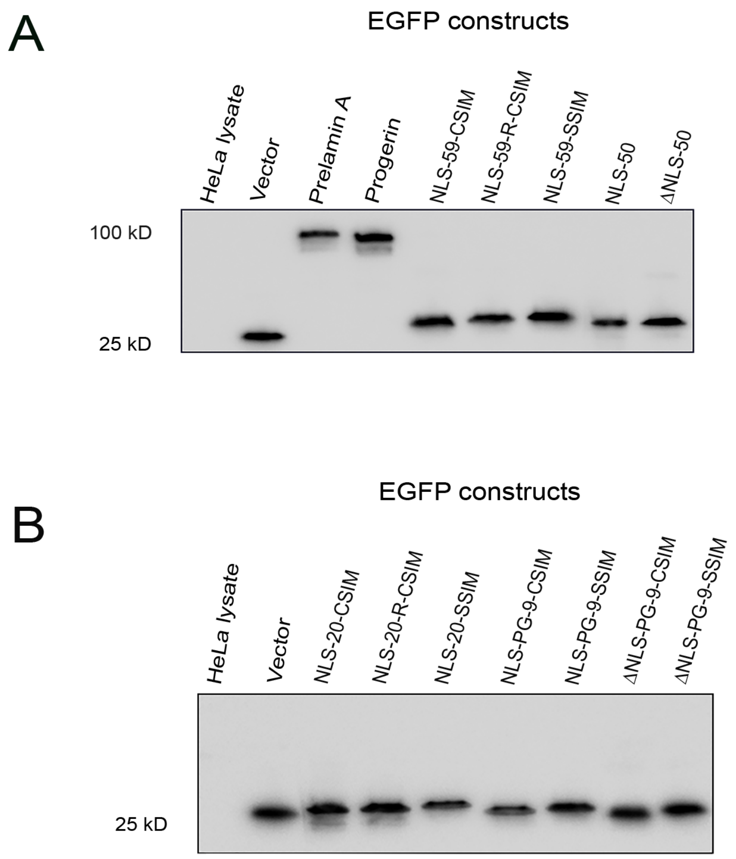

Figure S1. Western blot analyses of EGFP fusion proteins expressed in transiently transfected HeLa cells. Panels **A** and **B**: a HeLa cell lysate is used as a negative control. EGFPc1 empty vector-transfected cells are used as a second negative control to indicate the correct sizes of fusion proteins as indicated.

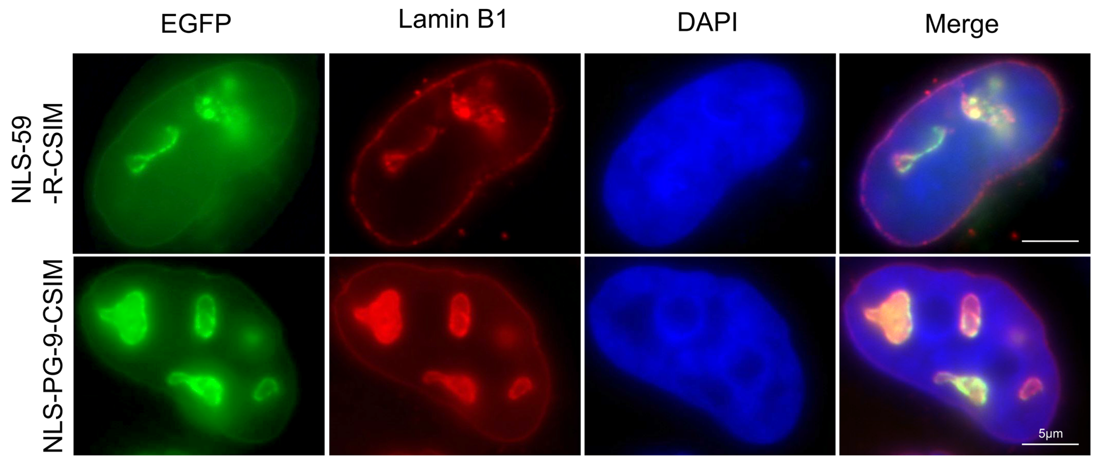

Figure S2. Abnormal nuclear envelope invaginations. Immunocytochemistry was performed in HeLa cells transfected with EGFP-NLS-59-R-CSIM and EGFP-NLS-PG-9-CSIM. Cells were stained with a lamin B1 antibody. Chromatin was stained with DAPI. Colocalization of EGFP-NLS-59-R-CSIM and EGFP-NLS-PG-9-CSIM with lamin B1 is shown. EGFP signals emanating from the nuclear membrane show abnormal nuclear invaginations. Scale bar, 10 μm.

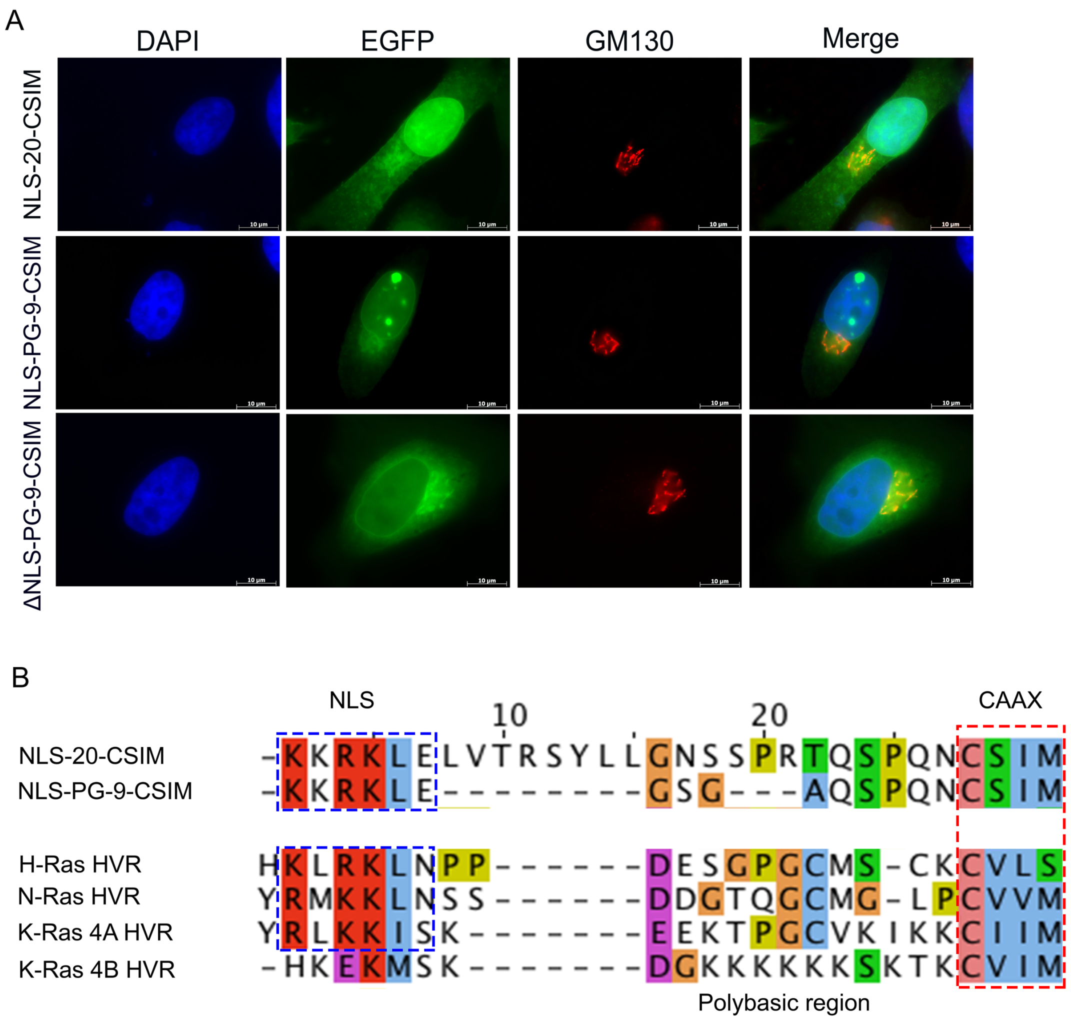

Figure S3. Trafficking of EGFP-NLS-20-CSIM and distribution of EGFP-ΔNLS-PG-9-CSIM. (**A**) GM130 was used to determine the cytoplasmic distribution of EGFP-NLS-20-CSIM signals. Representative images of cells expressing EGFP-NLS-20-CSIM are shown. The colocalization of cytoplasmic EGFP-NLS-20-CSIM with GM130 is shown. Chromatin was stained with DAPI. Scale bar, 10 μm. (**B**) An analysis of the similarity of the sequence of the NLS-20-CSIM proteins and hypervariable regions of RAS proteins, including H-Ras, N-Ras, K-Ras4A, and K-Ras4B, was performed. The NLS-20-CSIM protein showed a certain degree of similarity to the HVR of RAS proteins. Cellular distributions of RAS proteins are indicated by the rectangle. (C) Cells were stained with GM130 and calnexin to determine the distribution of EGFP-ΔNLS-PG-9-CSIM. Representative images of EGFP-ΔNLS-PG-9-CSIM-transfected cells show the accumulation of the protein at the Golgi apparatus, ER and outer nuclear membrane. Scale bar, 10 μm.

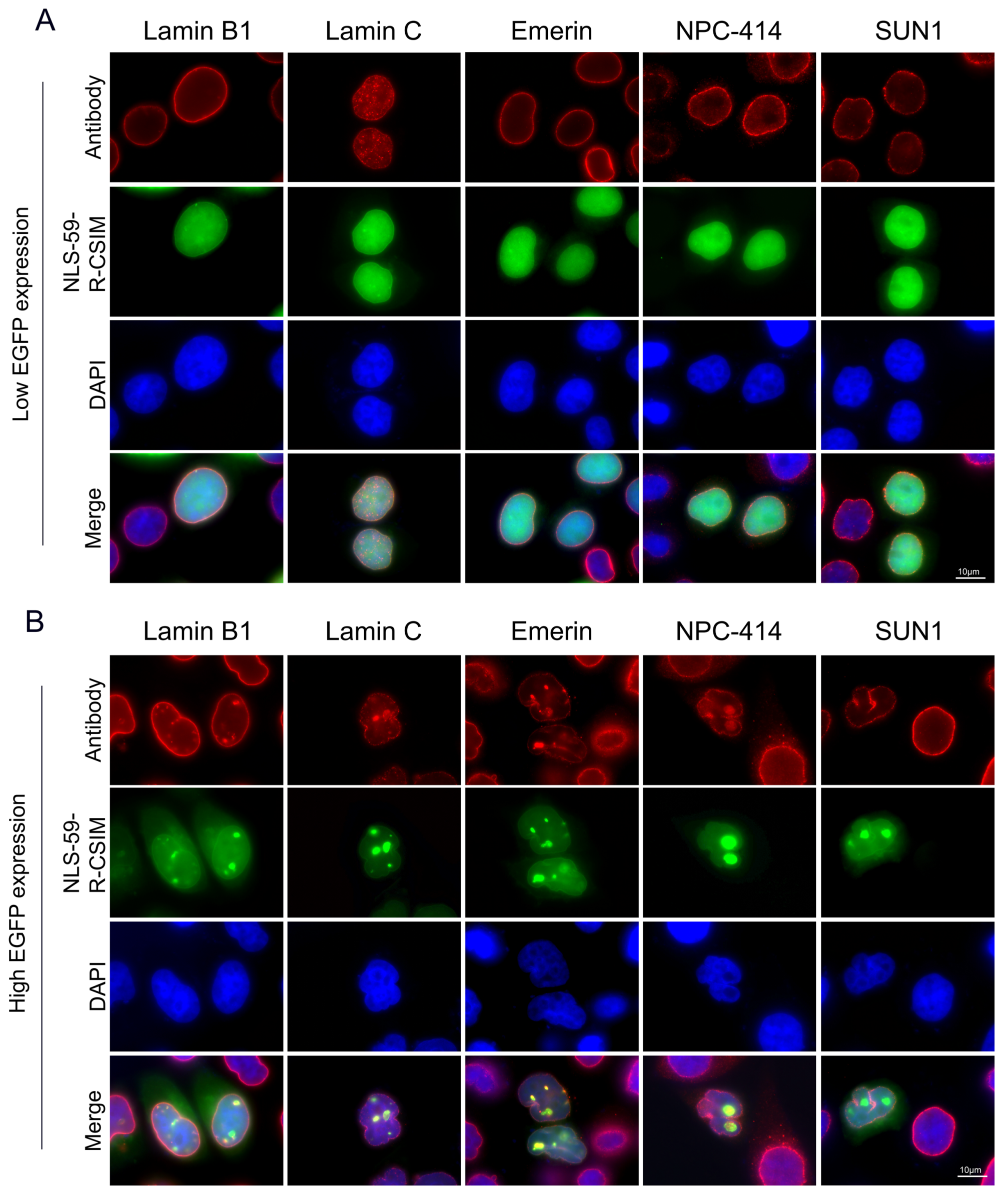

Figure S4. Concentration-dependent NE protein disorganization in EGFP-NLS-59-R-CSIM-expressing cells. Immunocytochemistry was performed on EGFP-NLS-59-R-CSIM-transfected HeLa cells. Cells were stained with lamin B1, lamin C, emerin, NPC-414, and SUN1 antibodies. Chromatin was stained with DAPI. Representative images of cells labeled for each protein are shown. Panel **A** shows cells with normal nuclear shapes and low EGFP signals. Panel **B** shows the co-localization of lamin B1, lamin C, emerin, and NPC-414 with EGFP signals at the NE, as EGFP-NLS-59-R-CSIM accumulates at the NE. SUN1 is absent from the NE invaginations. Scale bar, 10 µm.

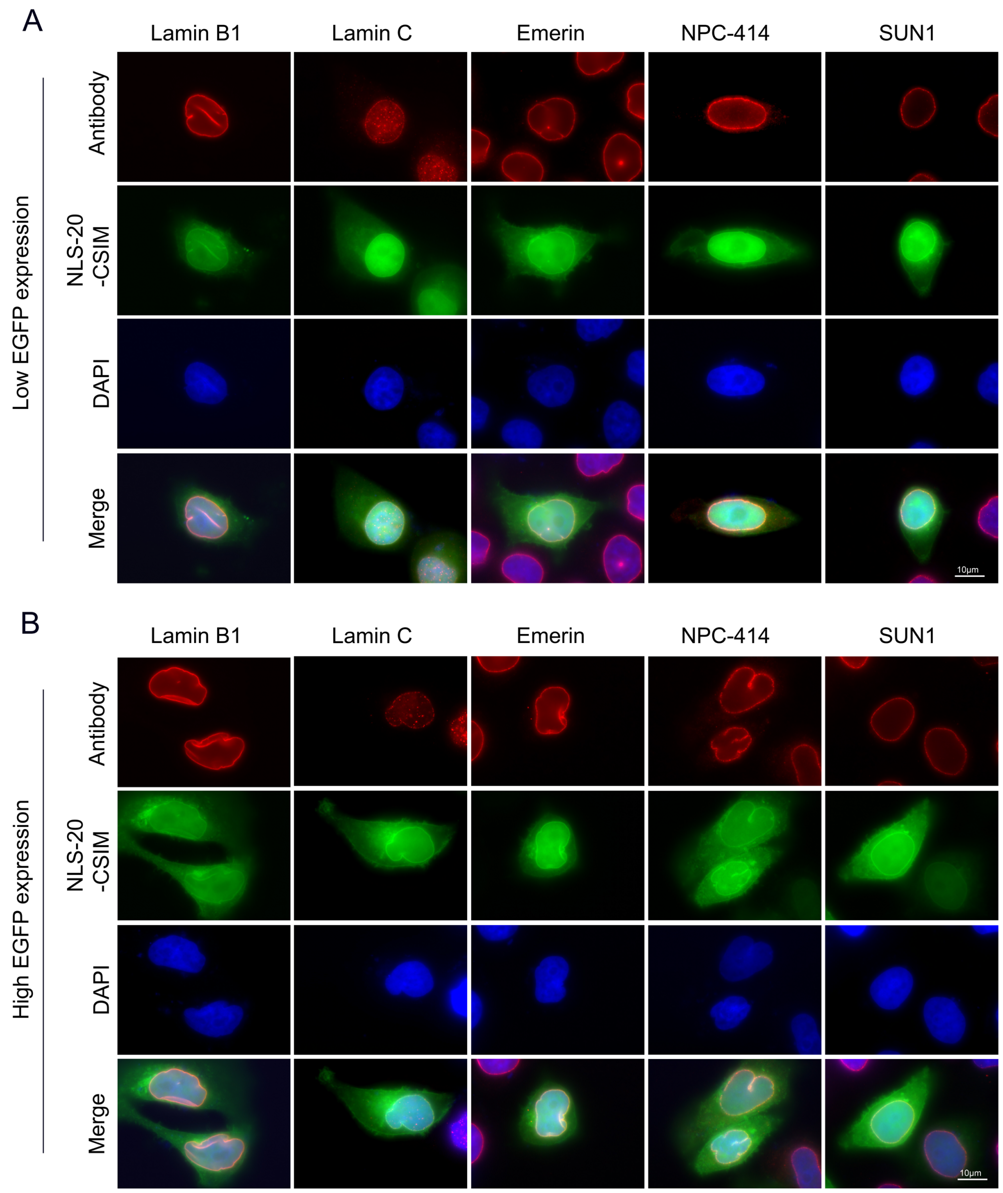

Figure S5. Concentration-dependent nuclear shape abnormalities in EGFP-NLS-20-CSIM-expressing cells. Immunocytochemistry was performed on EGFP-NLS-20-CSIM-transfected HeLa cells. Cells were stained with lamin B1, lamin C, emerin, NPC-414, and SUN1 antibodies. Chromatin was stained with DAPI. Representative images of the staining for each protein are shown. Panel **A** shows cells with normal nuclear shapes and low EGFP signals. Panel **B** shows cells with abnormal nuclear shapes, as EGFP-NLS-59-R-CSIM accumulates at the NE, cytoplasm and plasma membrane. Scale bar, 10 μm.

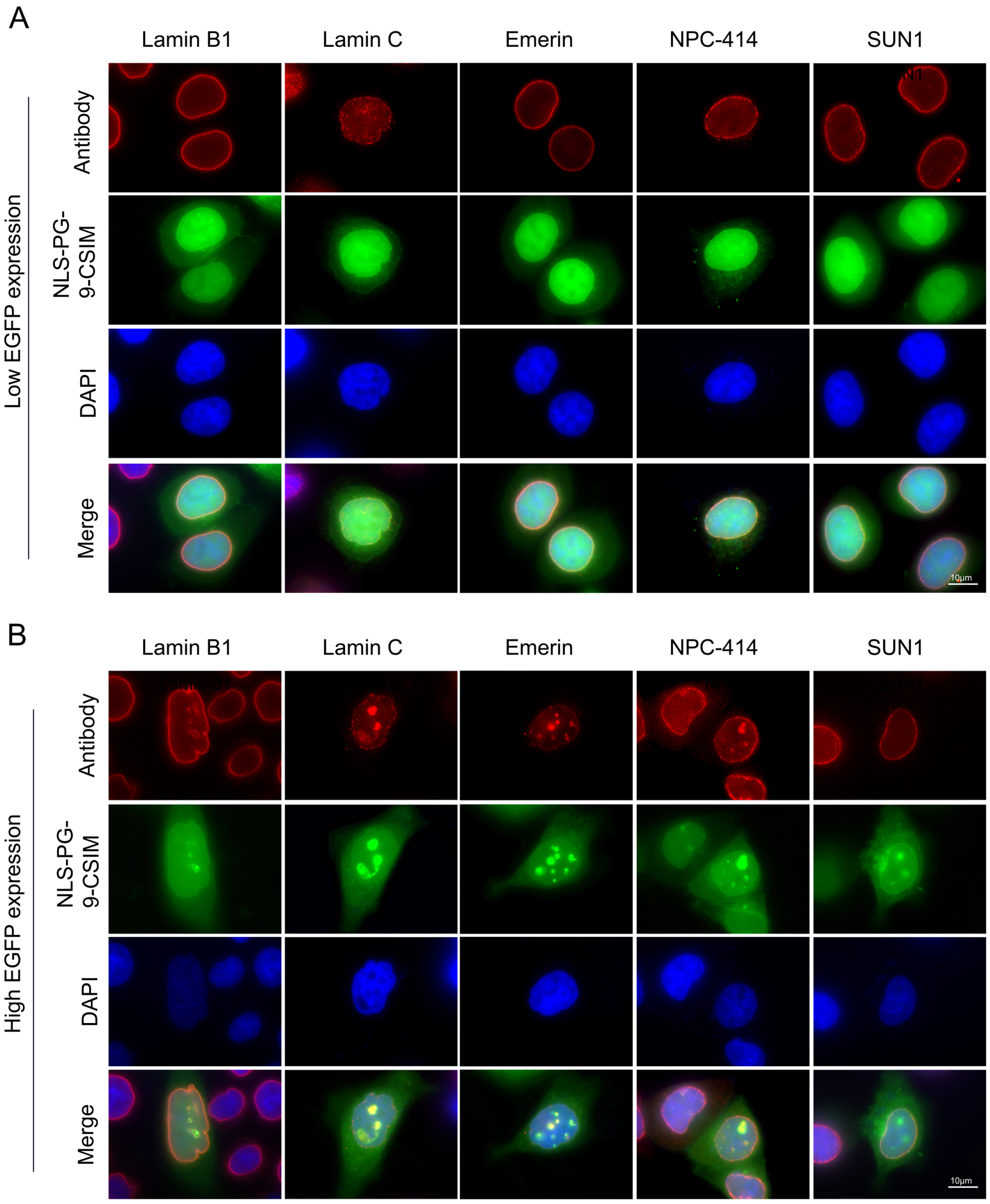

Figure S6. Concentration-dependent NE protein disorganization in EGFP-NLS-PG-9-CSIM-expressing cells. Immunocytochemistry was performed on EGFP-NLS-PG-9-CSIM-transfected HeLa cells. Cells were stained with lamin B1, lamin C, emerin, NPC-414, and SUN1 antibodies. Chromatin was stained with DAPI. Representative images of staining for each protein are shown. Panel **A** shows cells with normal nuclear shapes and low EGFP signals. Panel **B** shows the co-localization of lamin B1, lamin C, emerin, and NPC-414 with EGFP signals at the NE; EGFP-NLS-PG-9-CSIM accumulates at the NE. SUN1 is absent from the NE invaginations. Scale bar, 10 μm.

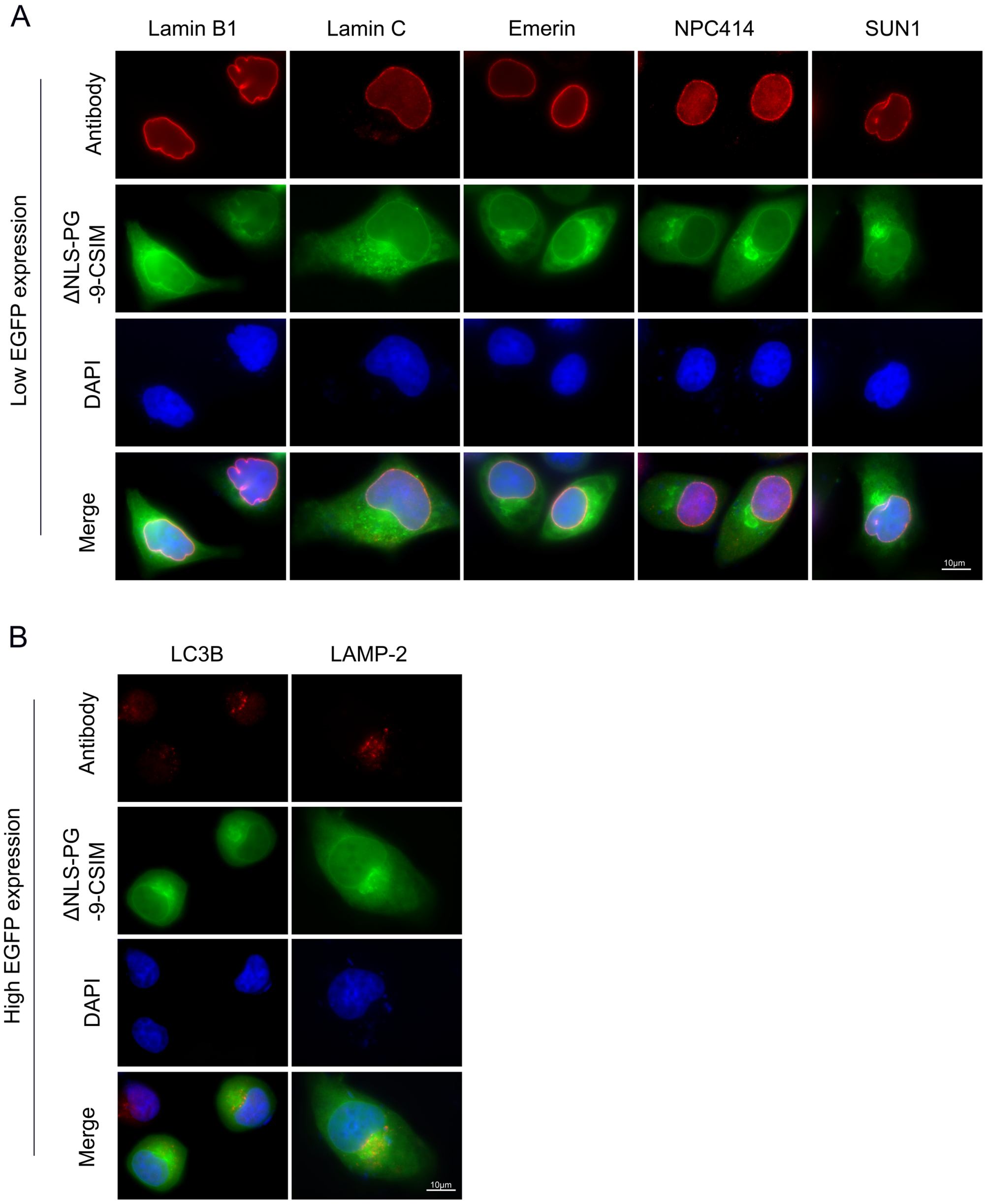

Figure S7. Immunodetection of NE proteins, cytoplasmic localization, and autophagosome-lysosome interactions in ΔNLS-PG-9-CSIM-expressing cells. (**A**) Immunocytochemistry was performed on EGFP-ΔNLS-PG-9-CSIM-transfected HeLa cells. Cells were stained with lamin B1, lamin C, emerin, NPC-414 and SUN1 antibodies. Chromatin was stained with DAPI. Representative images of staining for each protein are shown. (B) Cells were stained with LC3B and LAMP-2 antibodies to detect autophagosome-lysosome interactions with EGFP-ΔNLS-PG-9-CSIM in the cytoplasm. Scale bar, 10 μm.

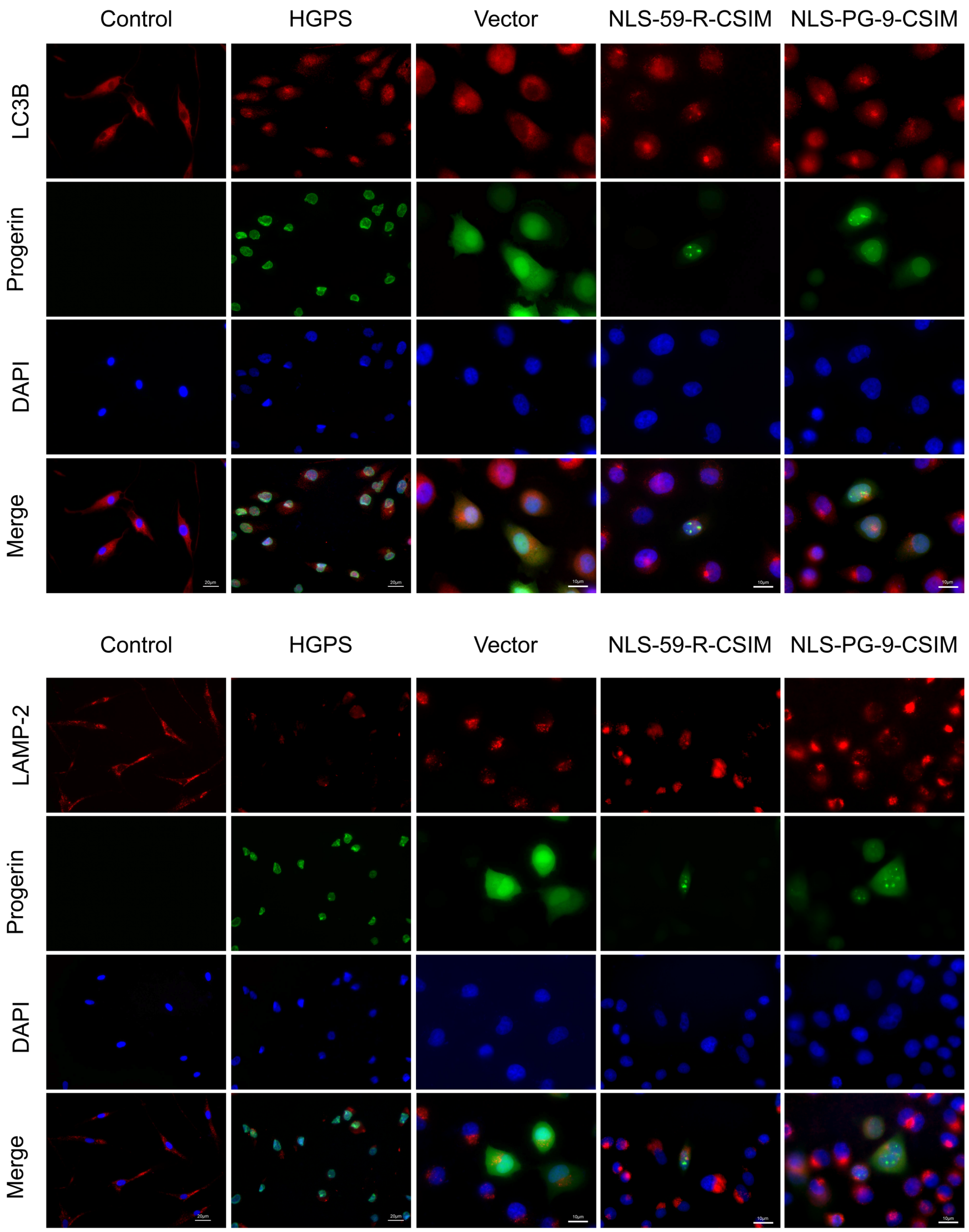

Figure S8. Autophagosome and lysosome distributions. (**A**) Immunocytochemistry was performed on primary fibroblasts from control subjects and patients with HGPS, as well as HeLa cells transfected with the empty vector, EGFP-NLS-59-R-CSIM or EGFP-NLS-PG-9-CSIM plasmids. Cells were stained with anti-LC3B and anti-progerin antibodies. Chromatin was stained with DAPI. Representative images of staining for each protein are shown. (**B**) Immunocytochemistry was performed on similar cells as indicated in (A) and probed with anti-LAMP2 and anti-progerin antibodies. Chromatin was stained with DAPI. Representative images of stained cells are shown. Scale bar, 10 μm.
